# An interview with Nadia Adotevi, 2023 *Epilepsia Open* Prize winner for basic science

**DOI:** 10.1002/epi4.12764

**Published:** 2023-06-07

**Authors:** Aristea S. Galanopoulou

**Affiliations:** ^1^ Saul R Korey Department of Neurology Dominick P Purpura Department of Neuroscience Isabelle Rapin Division of Child Neurology, Albert Einstein College of Medicine Beijing USA

## 1 PLEASE TELL US ABOUT YOURSELF

I am currently a postdoctoral researcher in the Department of Anesthesiology at the University of Virginia. I obtained my BSc (Hons) in Biological Sciences from the Kwame Nkrumah University of Science and Technology in Ghana, an MSc from Sheffield Hallam University, UK and a PhD from the University of Otago, New Zealand. After my PhD, I received postdoctoral training in the research laboratory of Dr. Jaideep Kapur (UVa Neurology), where this research was conducted.

## 2 HOW DID YOU BECOME INTERESTED IN CONDUCTING RESEARCH IN THIS FIELD?

During my studies, I became amazed by the complexity of brain structure and function and fascinated by how things could go wrong quickly. Seizures were a phenomenon that both scared and intrigued me. I first had the opportunity to do an epilepsy‐related research project characterizing hippocampal IP3R1 receptors during my Master's degree. I was able to continue epilepsy research during my PhD examining the cortical expression of AMPA receptors in an absence epilepsy model. Following my PhD graduation, I got the great opportunity to continue in this research field during my postdoctoral training in the Kapur laboratory.

## 3 PLEASE EXPLAIN THE QUESTION YOUR STUDY ADDRESSED, AND HOW YOU DESIGNED IT

Temporal lobe epilepsy (TLE) is the most common form of epilepsy. Many patients with TLE also report debilitating comorbidities including memory deficits, anxiety, and depression. The hippocampus is commonly implicated in seizure origination in patients with TLE. Nonetheless, focal resection of the hippocampus is not always successful in the treatment of TLE because seizure generation and spread could occur through the engagement of a vast brain network with multiple neuronal nodes. The interictal and ictal discharges impacting these extrahippocampal network nodes may be responsible for the associated comorbidities. However, a detailed description of this extensive neuronal network was unavailable, requiring the generation of neuronal activation maps with spatial resolution at the cellular level in experimental animals, which was performed in this study. A neuronal activation map aids the identification of brain structures and neuronal pathways involved in the progression of an ictal event from seizure onset to termination.

Employing electrical kindling, we recorded electrographic seizures from the hippocampi during a kindling‐evoked seizure and examined widespread activation patterns during these seizures in activity reporter TRAP2 mice. These transgenic mice exhibit permanent expression of Cre‐driven tdTomato in activated neurons in response to 4‐hydroxytamoxifen administration. Kindled animals exhibited behaviors including freezing, facial twitching, and involuntary head bobbing, similar to observations during human focal impaired awareness seizures of TLE. The expression of fluorescent reporters downstream of activity‐dependent promoters in these transgenic mice offer a much higher spatial and temporal resolution. We mapped the seizure circuits activated during these focal seizures using immunohistochemical approaches.

## 4 WHAT WERE THE RESULTS AND HOW DO YOU INTERPRET YOUR FINDINGS?

We provide a comprehensive brain‐wide mesoscale map of neuronal circuits preferentially activated during a focal hippocampal seizure in electrically‐kindled mice. The circuits mapped in this study are implicated in TLE as well as associated comorbidities such as memory deficits, depression, and anxiety disorders. We show significantly enhanced bilateral neuronal activation in multiple hippocampal, cortical, and thalamic structures during these seizures in contrast with previous studies showing limited activity during early seizure stages. There was bilateral seizure‐activation of circuits necessary for memory consolidation and those which regulate the stress response of hypothalamic–pituitary–adrenal axis. The hijack of these memory and depression regulatory systems by a focal hippocampal seizure could account for the frequent reports of comorbidities such as memory impairment, anxiety, and depression in many TLE patients.

## 5 WHAT ARE THE NEXT STEPS THAT YOU PLAN TO TAKE, AND WHAT ARE YOUR CAREER GOALS?

I have been immensely fortunate to have research experiences exploring multiple neurological disorders. My current research focus is on postoperative cognitive dysfunction (POCD) and neurodegeneration. I hope to further develop my research career by being able to incorporate all my skills, experiences, and collaborations into tackling confounding neurological questions that will translate into potential treatments and therapies for neurological diseases in the near future.

## 6 WHAT DOES THE *EPILEPSIA OPEN* PRIZE MEAN FOR YOU, YOUR LABORATORY, RESEARCH INSTITUTE, AND YOUR FUTURE?

I consider it a great privilege and honor to receive this award. It serves as a motivation for me to carry out my work with enthusiasm, passion, and diligence. I am grateful that this Prize provides an avenue for recognition and to highlight the epilepsy research being conducted in the Kapur lab to advance knowledge and provide new insights in this area. I would like to thank all past and present members of the lab, whom I have had the pleasure of working with, for their support. Ultimately, I hope that through this acknowledgment we can further develop our research and findings into translational research that subsequently improves health outcomes for patients with epilepsy and other neurological diseases.

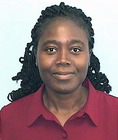

Read the winning article Focal impaired awareness seizures in a rodent model: a functional anatomy

